# Limb salvage in a partially amputated distal femur with extensive segmental bone loss using the nailing after lengthening technique: a case report

**DOI:** 10.1007/s11751-015-0212-8

**Published:** 2015-03-10

**Authors:** Gerald Eliot Wozasek

**Affiliations:** Department of Trauma Surgery, Medical University of Vienna, Waehringerguertel 18-20, 1090 Vienna, Austria

**Keywords:** Subtotal amputation with segmental long bone defect, Cement spacer, Bifocal lengthening, Secondary nailing

## Abstract

Segmental long bone defects resulting from high-energy trauma with severe soft tissue loss are difficult problems to manage. Amputation was for a long time the primary mainstay of treatment. This is the report on a 15-year-old male patient who sustained a third-degree open, traumatic fracture with partial amputation of the left distal femur and extensive bone loss of 26 cm. Successful limb salvage was performed after vascular repair, shortening of the bone defect, primary placement of an antibiotic cement spacer and simple external fixation. This was followed by bifocal lengthening modifying the simple frame until limb equality was achieved and secondary intramedullary nailing 11 months after injury.

## Introduction

Limb salvage in severe open injuries remains a challenge to orthopaedic surgeons. The decision for aggressive and heroic salvage against primary amputation still presents difficulty [1, 2]. When salvage is chosen, these injuries require a multidisciplinary approach, such as vascular reconstruction, free flaps with microsurgical techniques, advanced skeletal fixation and wound care therapies. The prolonged, demanding and controversial treatment may cause considerable mental and physical stress for the patient, and “successful” limb preservation can lead to functional deficits and secondary amputation.

The author presents a case of an open Gustilo type 3B fracture of the distal femur with an extensive segmental bone defect. The extent of injury and bone loss amounted to a near traumatic amputation. After vascular repair, the 26-cm bone defect was filled using a cement–Kirschner wire spacer and the limb was stabilized with a spanning external fixator. Restoration of anatomic integrity was achieved by bifocal lengthening using external fixation systems first and subsequently intramedullary (IM) nailing of the regenerated bone when leg length equality was achieved.

## Materials and methods

**Fig. 1:**
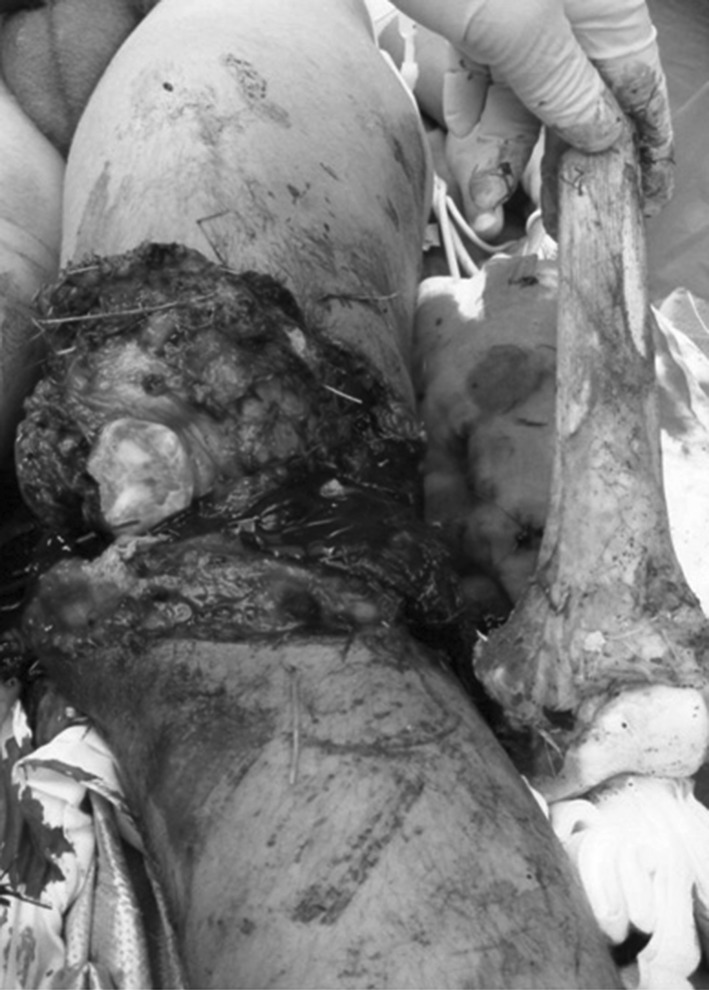
Third-degree open, subtotal amputation at the lower third of the left femur with severe soft tissue contamination. The distal part of the femoral bone was brought separately to the ER

**Fig. 2:**
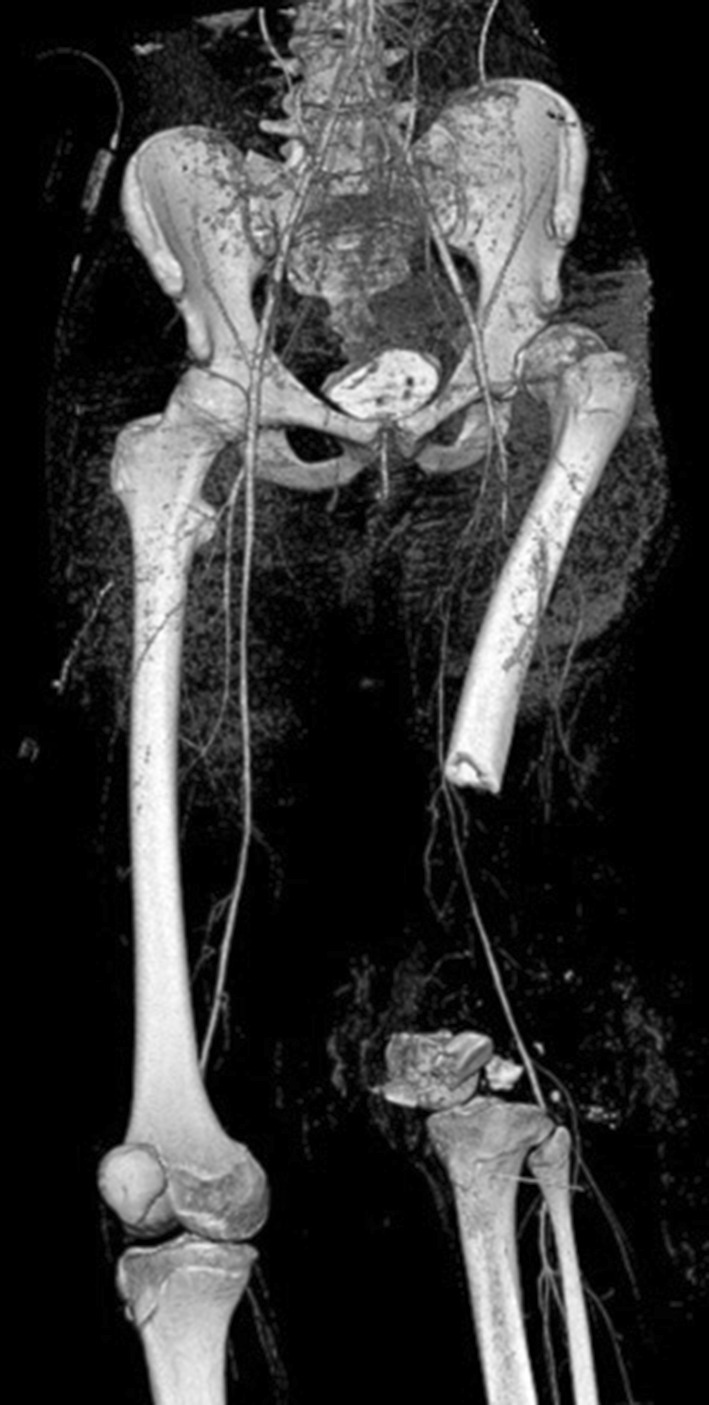
Computed tomography (CT) angiography demonstrates a complete dislocation of the left hip, segmental bone defect of approximately 26 cm of the distal femoral bone without signs of injury to the popliteal artery

**Fig. 3:**
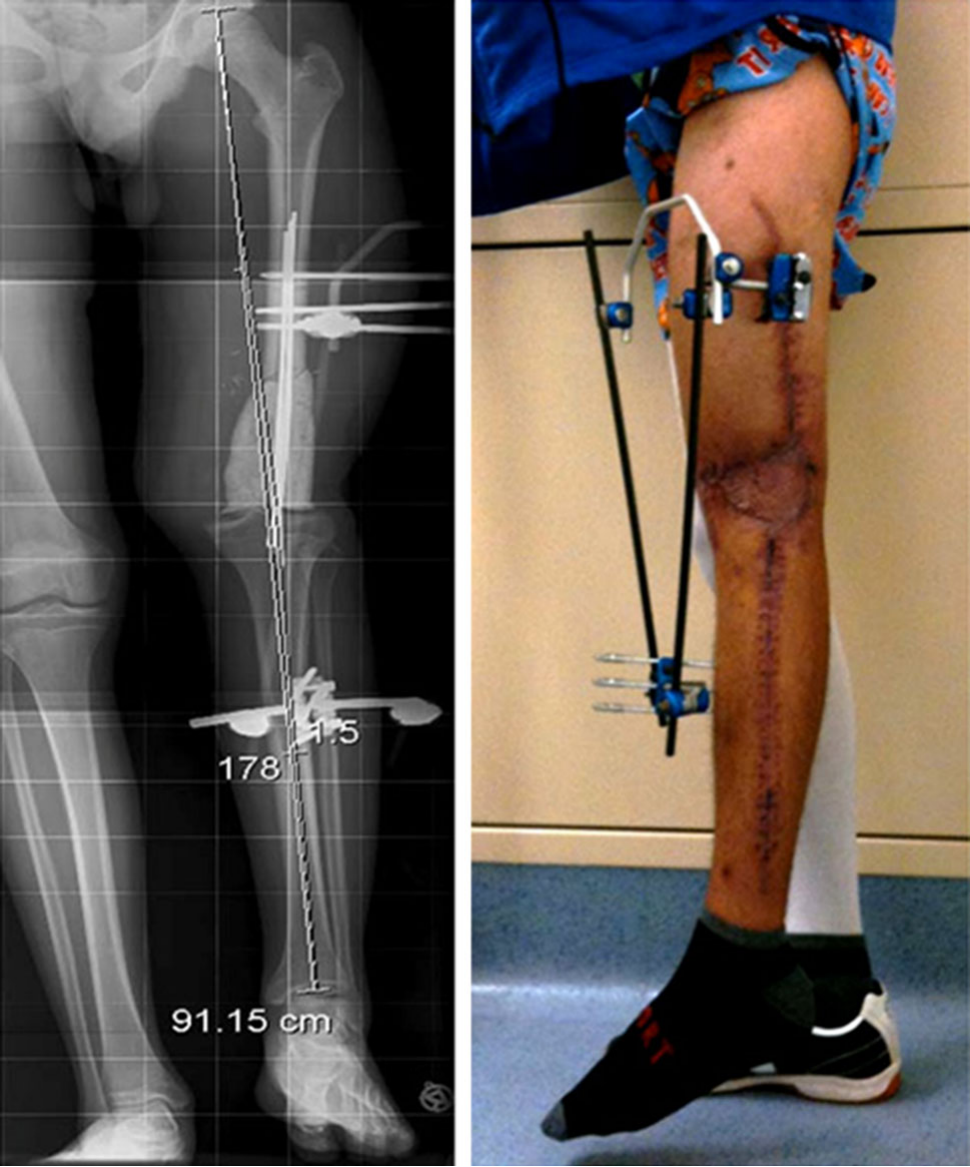
Clinical and radiological findings 6 weeks after injury

A 15-year-old boy sustained injuries in a motor cycle accident and was intubated at the scene before transfer by emergency helicopter to our Level I trauma centre. On arrival in the emergency room (ER), he presented with stable cardiopulmonary vital signs, normal pupil reaction and no signs of injuries to the upper extremity, abdomen or pelvis. Standard radiograph of the chest revealed a pneumothorax of the right lung for which a chest tube was placed. Clinical examination of the lower extremity showed a subtotal amputation of the left distal femur (AO 43-C3) with extensive soft tissue loss and wound contamination. The distal third of the femur was retrieved from the scene of the accident and brought separately to the ER (Fig. 1). Standard radiographs revealed in addition a dislocation of the left hip. Broad-spectrum antibiotic treatment was initiated immediately. The dorsalis pedis artery could not be palpated; computed tomographic (CT) angiography revealed an extensive bone defect of approximately 26 cm of the distal third of the left femur, no injury to the femoral artery but an injury to the femoral vein (Fig. 2). Clinical neurological examination was not possible. A decision for attempted limb salvage was made taking into consideration the patient’s age and the absence of injury to the femoral artery. Within 1 h of ER admission, the patient was brought to the operating theatre and the hip dislocation reduced. A thorough debridement of all contaminated and necrotic soft tissue was then performed. The popliteal area was explored confirming no damage to the popliteal artery and nerve. Surgical repair of the femoral vein was performed by a vascular surgeon. The distal part of the femur and proximal part of the tibia were shortened and connected with a k-wire and bone cement spacer (Palacos^®^, Zimmer) with gentamicin added, creating a solid interposition construct (Fig. 3). A complete fasciotomy was performed and a synthetic skin replacement (Epigard^®^, Consept GmbH) as temporary wound dressing applied. In addition, a spanning external fixator was mounted for skeletal stabilization of the limb.

**Fig. 4:**
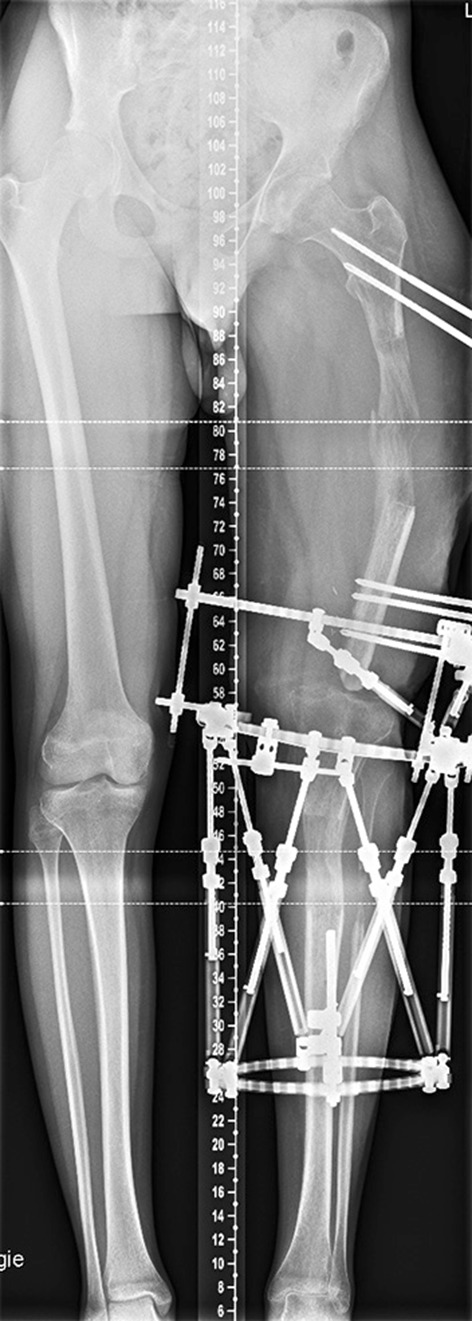
Radiological findings 250 days after injury preoperatively before nailing procedure

**Fig. 5:**
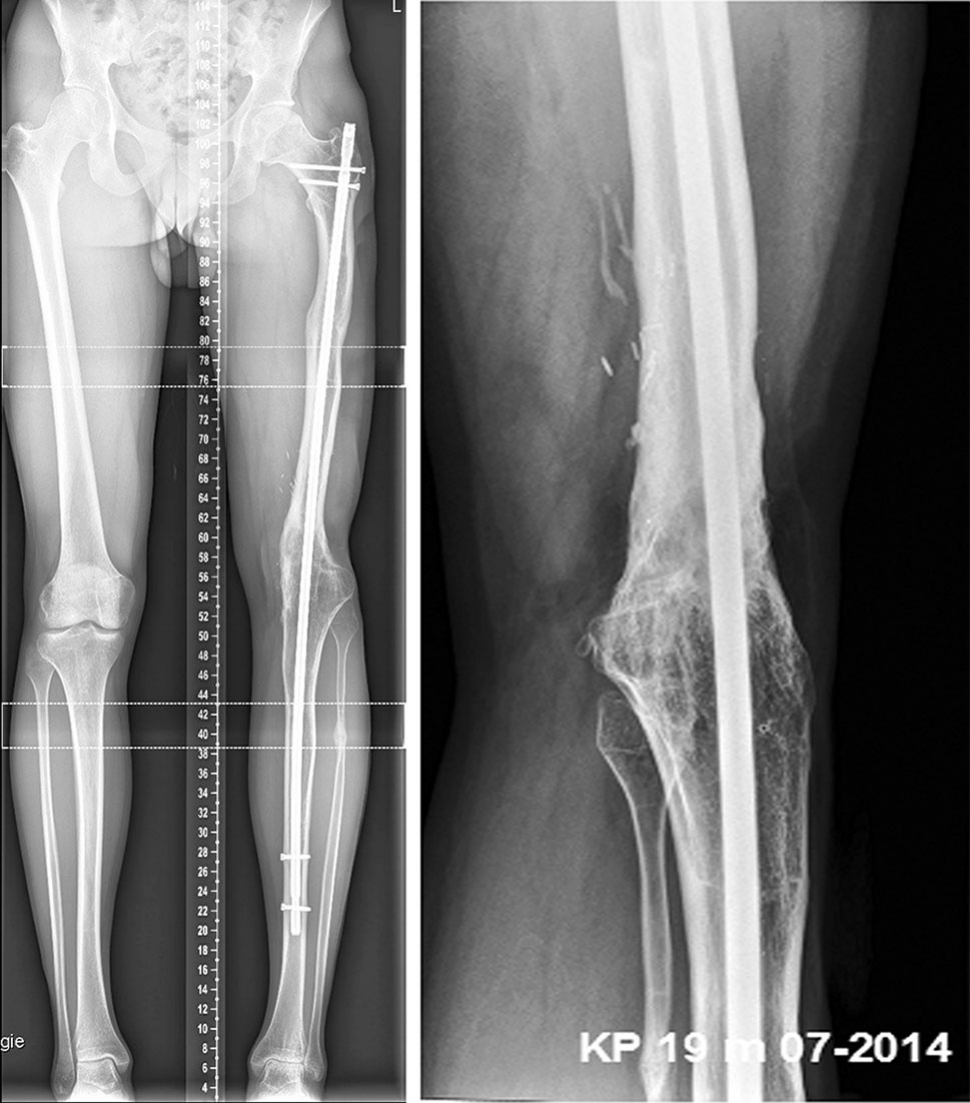
Radiological findings after 2 years with solid bony union

**Fig. 6:**
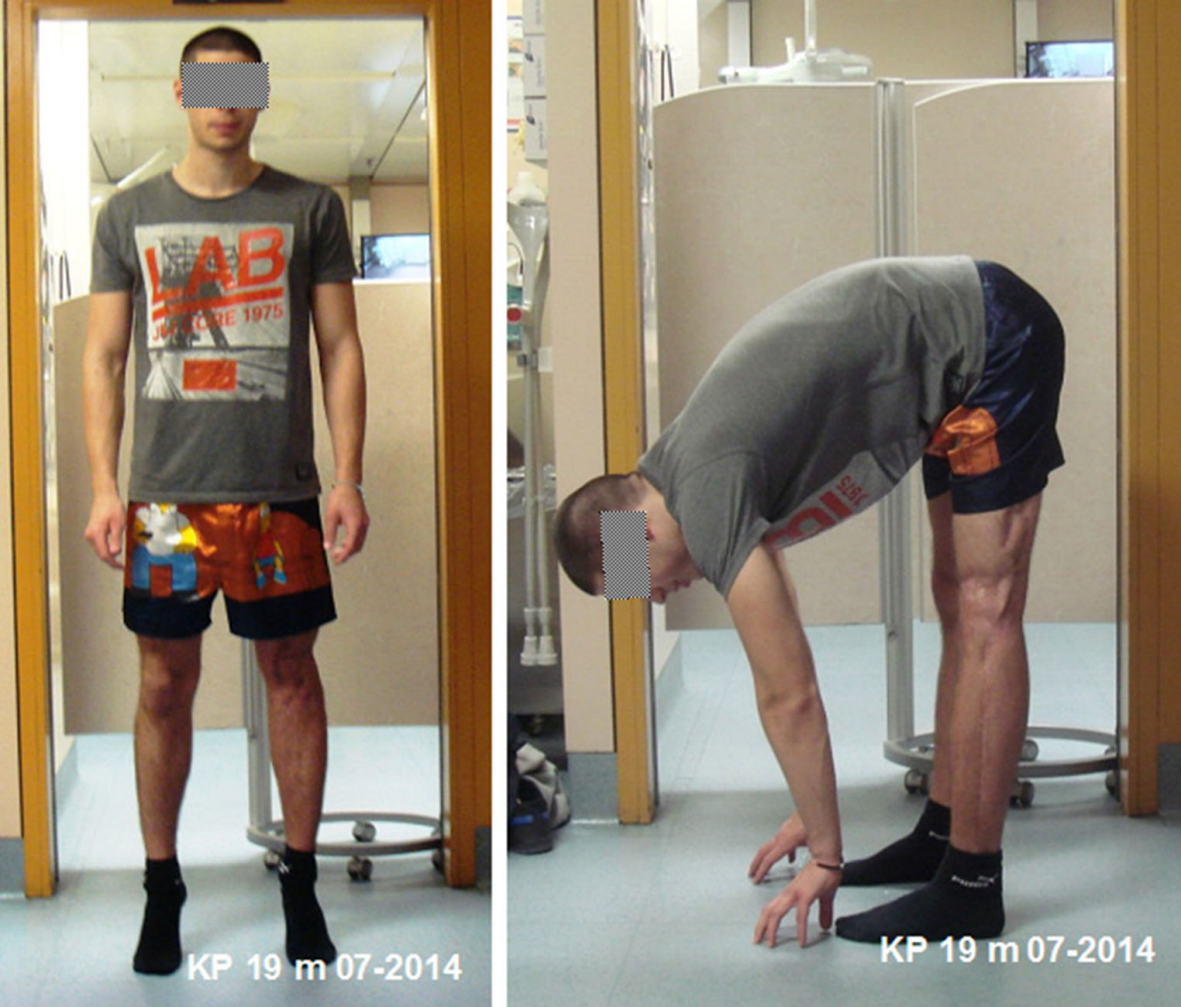
Clinical findings after 2 years shows good functional result

Anteroposterior and lateral radiographs after surgery revealed an appropriate position for the bone cement spacer. The Epigard cover was changed every 2 days until the wound was clean and able to be covered by meshed skin grafting (Fig. 3). A long-cassette radiograph and clinical evaluation revealed a limb length difference of 10 cm with the bone cement spacer in situ. Bifocal bone lengthening of both the femur and tibia was planned due to the total length of the bone defect. A mono-lateral external fixator (Monotube™, Stryker) was assembled at the femur, and an osteotomy of the proximal femoral bone was performed. For the tibia, a Taylor Spatial Frame (TSF) (Smith & Nephew, Inc., Memphis, TN) external ring fixator was mounted followed by an osteotomy of the proximal tibia and fibula for bifocal callus distraction at both the femoral and tibial sides. Distraction was started on the sixth post-operative day, and the rate of callus lengthening was individualized according to the regenerate appearance on radiographs which were performed every 2 weeks during the distraction period (Fig. 4). The patient was mobilized with partial weight bearing permitted. At 3 months, the bone cement spacer and k-wires were removed and a local antibiotic chain (Septopal^®^, Biomet, Warsaw, Indiana, USA) with 30 beads implanted into the bone defect. The chain was extracted percutaneously gradually over 10 days. After 8 months (250 days) and a total callus distraction length of 16 cm at the femoral and 9.8 cm at the tibial side, radiographs and clinical evaluation revealed limb length equality (distraction index, 11.46 day/cm). The external fixation system was removed, and a reamed knee arthrodesis intramedullary nail (T2^®^, Stryker) was inserted aiming to stabilize the newly formed regenerate from bending or re-fracture. At 4 months following IM nailing, standard radiographs displayed a non-union at the docking site of the distal part of the femoral segment. A decision to bone-graft the non-union was taken and autologous bone graft retrieved through the contralateral femur via the greater trochanter, utilizing the Reamer–Irrigator–Aspirator (RIA) device (Synthes, Pennsylvania, USA). The autologous bone graft was combined with bone morphogenetic protein 7 (BMP-7) (Osigraft, Stryker, UK) and packed to fill the non-union. The subsequent recovery was uneventful, and complete bone healing was achieved after 2 years (Fig. 5). At the final clinical assessment of 4 years following the end of the limb reconstruction treatment, the patient showed good functional results (Fig. 6).

## Discussion

The debate continues over to perform primary amputation or limb salvage in a high-energy, complex open fracture with extensive bone defect in the lower extremities [3]. Both strategies have advantages and disadvantages. Aside the functional loss, body image and mental health issues after primary amputation surgery, patients can be at risk for recurring stump problems, phantom pain and discomfort in prosthesis [4, 5]. Limb salvage requires a multidisciplinary approach including repeated surgeries from orthopaedic, plastic and vascular surgeons, with long hospitalization and recovery periods adding to high costs [4, 6].

The Ilizarov method of distraction osteogenesis has been developed for the treatment of large segmental bone defects and allows salvage of lower extremity injuries that may otherwise have been amputated [7–9]. Despite the potential for success, callus distraction is time consuming and has a high complication rate such as pin tract infection, pain and the personal discomfort associated with the external fixator system [10]. After removal of the external frame during the consolidation phase, the newly regenerated soft callus is at increased risk of re-fracture and bending due to the lack of sufficient strength. In order to avoid these potential complications, the external frame was removed and the limb and new regenerate stabilized with an intramedullary nail as soon as limb length equality was achieved, according to the nailing after lengthening (NAL) procedure [11]. This treatment concept has the additional advantage of reducing the period in the frame and returns the patient’s mobility, comfort and activity level earlier.

The use of a cement spacer filled within the extensive bone defect has been originally described by Masquelet [12] in the context of the “induced membrane technique” for the treatment of bone defects. In this case, an antibiotic cement cylinder was moulded around multiple interposed k-wires within the defect. The spacer avoids soft tissue collapse, creates a soft tissue tunnel for later reconstruction, and maintains the soft tissue anatomy whilst delivering a high local dose of antibiotic.

There were no signs of deep infection after 4 years of follow-up. We used established principles of reconstruction including initial thorough debridement, the antibiotic/cement spacer, delayed reconstruction using distraction osteogenesis and subsequent arthrodesis with an intramedullary nail to success. Nevertheless, a 4-year follow-up is limited in terms of declaration of being free of infection and longer-term follow-up is needed.

A number of scoring systems (e.g. MASS—mangled extremity severity score, LSI—limb salvage index) have been utilized to make an objective assessment in the context of limb salvage versus primary amputation in limb-threatening injuries. For this case, there is little doubt that early amputation would have allowed quicker recovery, shorter hospitalization and possible lower costs in the short-to-medium term. However, modern sophisticated limb prostheses are expensive and require several replacements during the course of a patient’s lifetime. In our opinion, immediate amputation is indicated as a means for haemorrhage control or in cases of severe crush injuries. The size of a bone defect, whilst important, is not an absolute indication, and options for reconstruction of large diaphyseal bone defects include biologic and non-biologic materials. In younger patients, biologic reconstruction is favoured and includes techniques such as vascularized bone graft. This is demanding technically, and there are potential complications including a high incidence of stress fractures, malunion and donor-site morbidity. In our patient, it would be unlikely for a transplanted fibula to have enlarged sufficiently in the width to equal the missing distal femoral bone. In adolescent patients, limb salvage using a staged treatment protocol is an appropriate alternative strategy, but the decision is one of clinical judgment and expertise of the orthopaedic surgeon and multidisciplinary team.

The principles of limb salvage and reconstruction were applied in this patient. The initial surgery was to set a foundation for reconstruction: debridement, venous vascular repair, shortening of the limb, placement of a wire and bone cement spacer into the defect, synthetic skin graft and temporary simple frame fixation. When wound closure was achieved, leg length discrepancy was treated by bifocal external callus distraction. In the last step, the arthrodesis nail was implanted to reduce the duration of the frame and to correct the limb alignment.

In conclusion, limb preservation in high-energy complex fractures of the lower extremities with extensive bone loss and soft tissue compromise is a costly and time-consuming procedure that requires a multidisciplinary approach. The limb should be considered for salvage, especially in young and active patients who are mentally prepared and willing to spend the time in the external fixator and undergo the multiple surgeries. This particular case of 26 cm of traumatic bone loss is unusual but highlights how principles including the Masquelet cement spacer, Ilizarov distraction osteogenesis and the NAL can be made to work together for successful limb salvage.
